# Renal Microcirculation and Calcium Channel Subtypes

**DOI:** 10.2174/1573402110666140131160617

**Published:** 2013-08

**Authors:** Koichiro Homma, Koichi Hayashi, Shintaro Yamaguchi, Seitaro Fujishima, Shingo Hori, Hiroshi Itoh

**Affiliations:** Department of Internal Medicine, School of Medicine, Keio University;; Department of Emergency and Critical Care Medicine, School of Medicine, Keio University, Tokyo, Japan

**Keywords:** Afferent arterioles, efferent arterioles, glomerular pressure, renal injury.

## Abstract

It has recently been reported that voltage-dependent Ca channel subtypes, e.g., L-, T-, N-, and P/Q-type, are expressed in renal arterioles and renal tubules, and the inhibition of these channels exerts various effects on renal microcirculation. For example, selective blockade of L-type Ca channels with nifedipine preferentially dilates the afferent arteriole and potentially induces glomerular hypertension. On the other hand, recently developed Ca channel blockers (CCBs) such as mibefradil and efonidipine block both T-type and L-type Ca channels and consequently dilate both afferent and efferent arterioles, leading to lowering of intraglomerular pressure. Interestingly, aldosterone has recently been recognized as a factor exacerbating renal diseases, and its secretion from adrenal gland is mediated by T-type Ca channels. Furthermore, T-type CCBs were shown to ameliorate renal dysfunction by suppressing inflammatory processes and renin secretion. On the basis of histological evaluations, N-type Ca channels are present in peripheral nerve terminals innervating both afferent and efferent arterioles. Further, it was suggested that N-type CCBs such as cilnidipine suppress renal arteriolar constriction induced by enhanced sympathetic nerve activity, thereby lowering intraglomerular pressure. Taken together, various Ca channel subtypes are present in the kidney and blockade of selective channels with distinct CCBs exerts diverse effects on renal microcirculation. Inhibition of T-type and N-type Ca channels with CCBs is anticipated to exert pleiotropic effects that would retard the progression of chronic kidney disease through modulation of renal hemodynamic and non-hemodynamic processes.

## INTRODUCTION

The kidney receives a large amount of blood supply (approximately 20–30% of the cardiac output) and maintains water-electrolyte balance through the processes such as glomerular filtration and tubular reabsorption. In addition, the kidney is a target organ in a variety of diseases such as hypertension and diabetes. It has been established that renin–angiotensin system inhibitors such as ACE inhibitors and angiotensin receptor blockers (ARBs) offer renal protective action. Furthermore, Ca channel blockers (CCBs) are widely used as antihypertensive agents in the clinical setting in which renal function is impaired. Recent advances in clinical pharmacology demonstrate that the conventional CCBs, including nifedipine and amlodipine, cause a large increase in glomerular filtration rate (GFR) as well as an elevation in filtration fraction, an indicator of glomerular pressure [1, 2]. This observation suggests that these CCBs predominantly dilate preglomerular (i.e., afferent) arterioles. GFR is primarily regulated by glomerular hemodynamics and the balance between afferent and efferent arterioles adjoining the glomerulus. To the extent that GFR is maintained at a constant level in the face of altered renal perfusion pressure [3], it is conjectured that the afferent and efferent arteriole are controlled by different vasomotor mechanisms. In fact, afferent and efferent arterioles differ with respect to the histological features of their respective cell components; at the molecular level, the vascular smooth muscle cells of afferent and efferent arterioles possess different myosin heavy chain isoforms [4]. The divergent profiles of these arterioles may also be related to the distribution of Ca channels in the renal microvasculature. 

## CALCIUM CHANNEL SUBTYPES

**Fig. (1):**
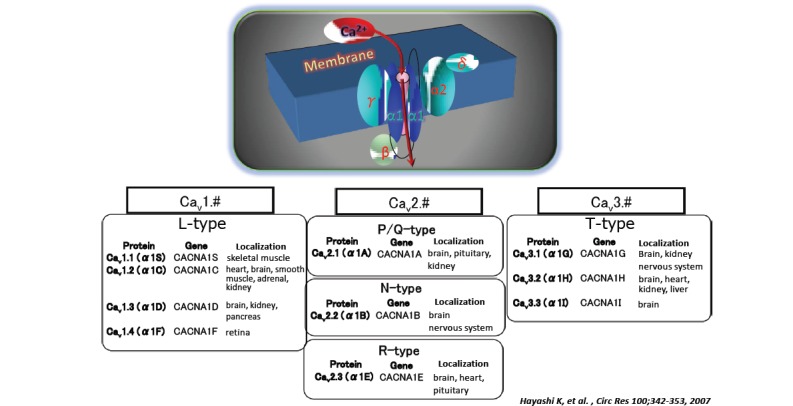
Ca2 channel structure and nomenclature.

Voltage-dependent Ca channels are be classified by their electrophysiological and pharmacological properties into 2 categories; high voltage-dependent Ca channels, which include L-, P/Q-, N- and R-types Ca channels, and low voltage-dependent Ca channels, named T-type Ca channels. Many high voltage-dependent Ca channels are functional as tetramers of α1, α2δ, β and γ subunits (Fig. **1**) [5]. Among those, an α1 subunit constitutes a principal component that forms pores through which Ca^2+^ enters into the intracellular space, whereas the remaining subunits inhibit channel function as regulatory or accessory subunits. In contrast, T-type Ca channels are considered functional as a single α1 subunit, although the functional involvement of accessory subunits has not been clarified thus far. In the kidney, a variety of α1 subunits are expressed, including Ca^2＋^V2.1 (α1A), Ca^2＋^V1.2 (α1C), Ca^2＋^V1.3 (α1D), Ca^2＋^V3.1 (α1G) and Ca^2＋^V3.2 (α1H), and these subunits function as L-type (Ca^2＋^V1.2, Ca^2＋^V1.3), T-type (Ca^2＋^V3.1, Ca^2＋^V3.2) and P/Q-type (Ca^2＋^V2.1) channels [5]. Additionally, the kidney is densely innervated and N-type Ca channels are present at the nerve terminals. 

## CA CHANNEL SUBTYPES AND NEPHROPATHY

### Role of Ca Channel Subtypes in Renal Arterioles

**Fig. (2):**
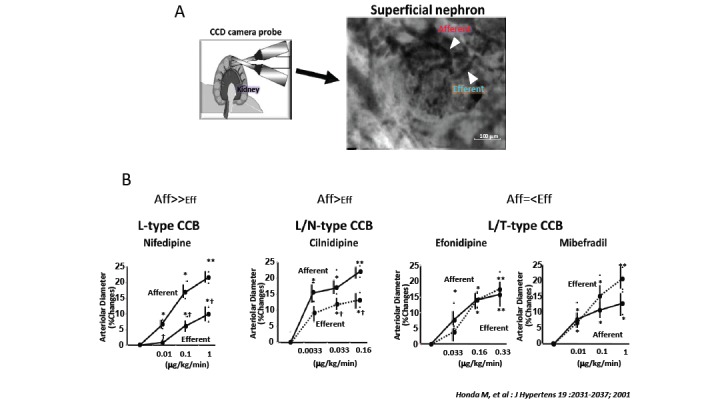
**Effects of various Ca2 channel blockers on in vivo renal microvessels and renal hemodynamics. A**, Direct in vivo visualization of renal microcirculation with the use of intravital pencil-type charge-coupled device videomicroscopy. **B**, CCBs with preferential blockade of L-type Ca2 channels cause predominant afferent arteriolar action (nifedipine), whereas CCBs with blocking activity on L-/T-type Ca2 channels dilate both afferent and efferent arterioles (efonidipine and mibefradil). Cilnidipine with L-/N-type Ca2 channel–blocking action dilates both microvessels, although the response is greater in the afferent arteriole. #P=0.05 vs baseline, *P<0.05 vs baseline, **P<0.01 vs baseline, §P<0.05 vs nifedipine, †P<0.05 vs afferent arterioles.

The progression of nephropathy is largely influenced by intraglomerular pressure, which is controlled by the balance between the afferent and efferent arteriole adjoining the glomerulus. L-type Ca channels have been shown to be present in the afferent arteriole, whereas T-type Ca channels are prevalent in both afferent and efferent arterioles [6]. The heterogeneity in the distribution of Ca channel subtypes would result in the consequence that the blockade of L-type Ca channels dilates afferent arterioles whereas the inhibition of T-type Ca channels dilates both afferent and efferent arterioles. Indeed, using an experimental system of the isolated hydronephrotic kidney, we found that efonidipine and benidipine, T-type CCBs, dilated efferent as well as afferent arterioles [7]. In contrast, CCBs with only inhibitory activity on L-type Ca channels such as nifedipine and amlodipine elicited predominant dilation of afferent arterioles [7]. Furthermore, the same results were obtained using an *in vivo* charge-coupled device (CCD) camera method, an experimental system that can evaluate renal arteriolar responses under almost physiological conditions (Fig. **2**) [8]. 

Experimental systems used for observation of the renal microcirculation are divided into two types based on the presence or absence of neuronal activity. Thus, no neuronal activity is observed in the isolated hydronephrotic kidney model whereas this can be visualized in the *in vivo* CCD camera method [5]. Because both afferent and efferent arterioles are innervated and N-type Ca channels are present in peripheral nerve terminals, CCBs with inhibitory activity on N-type Ca channels are anticipated to dilate both arterioles through the inhibition of sympathetic activity, which can be visualized in the in the vivo CCD camera method, but not in the isolated hydronephrotic kidney model [5]. 

### Diverse Inhibition of Ca Channel Subtypes by CCBs

A large number of dihydropyridine Ca channel blockers have been developed, of which nifedipine and amlodipine are representative. It has been well established that CCBs act on voltage-dependent Ca channels, specifically of L-type Ca channel subtypes. Additionally, evidence has been accumulated that several CCBs inhibit not only L-type Ca channels but also other subtypes of Ca channels [9]. Efonidipine, benidipine and mibefradil are reported to inhibit T-type Ca channels as well as L-type Ca channels. Furthermore, cilnidipine suppresses Ca signals through the inhibition of both L-type and N-type Ca channels. 

### Effects of CCBs on Glomerular Hemodynamics

The findings on intrarenal distribution of Ca channel subtypes and the action of various CCBs on these channels suggest divergent changes in intraglomerular pressure by CCBs. Thus, L-type CCBs is anticipated to increase intraglomerular pressure because of their preferential afferent arteriolar dilation and consequently the direct transmission of systemic blood pressure, unless systemic blood pressure is sufficiently reduced. In contrast, T-type and N-type CCBs are not considered to increase or rather decrease intraglomerular pressure because they dilate both afferent and efferent arterioles [8]. Concordantly, the filtration fraction, which approximately parallels the changes in intraglomerular pressure, reveals an increase by an L-type CCB nifedipine, no change by an N-type CCB, cilnidipine, and a decrease by T-type CCBs, mibefradil and efonidipine [10]. These changes in intraglomerular pressure would affect urinary protein excretion and the development of nephro-pathy. Fujiwara *et al*. [11] demonstrated that nifedipine, efonidipine and enalapril decreased blood pressure in a similar manner in a rat chronic kidney disease (CKD) model; the increase in urinary protein excretion, however, was significantly higher with nifedipine than with efonidipine and enalapril. Furthermore, these results were consistent with the renal histological findings. 

Evidence been accumulated showing aldosterone exerts renal hemodynamic action. Arima *et al*. [12] examined the effects of aldosterone on renal arteriolar tone. They demonstrated aldosterone is a potent vasoconstrictor on both afferent and efferent arterioles, which potentially leads to intraglomerular hypertension. Based on the observation that spironolactone reduces albuminuria without changes in blood pressure in diabetic patients treated with ACE inhibitors [13], aldosterone would cause intraglomerular hypertension *in vivo*. In this regard, efonidipine is demonstrated to dilate the aldosterone-induced constriction of both afferent and efferent arterioles [12]. Furthermore, T-type, but not L-type, CCBs are reported to inhibit aldosterone synthesis and release in cultured adrenal cells [14, 15]. Actually plasma aldosterone levels are lower in patient treated with T-type CCBs than in those treated with L-type CCBs [16, 17]. Collectively, these observations lend support to the premise that T-type CCBs offer additional benefits in aldosterone-mediated renal injury. 

Increases in sympathetic nerve activity are a matter of interest in the pathophysiology of CKD. The sympathetic nerve terminal is present in both afferent and efferent arterioles; increased sympathetic nervous activity augments vascular resistance and intraglomerular pressure. Actually, a centrally acting sympathetic nerve inhibitor moxonidine reduces albuminuria in hypertensive patients with microalbuminuria [18] and in a rat 5/6 nephrectomy model [19]. Of interest, an increasing number of studies with azelnidipine assess its effect on the sympathetic nervous system. Although CCBs generally increase pulse rate because of reflex hyperactivity countering the reduction in blood pressure, azelnidipine has been shown to reduce pulse rate [20]. In a study using spontaneously hypertensive rats, azelnidipine was shown to reduce heart rate by inhibiting sympathetic nerve activity [21]. Additionally, azelnidipine was shown to reduce intraglomerular pressure by dilating both afferent and efferent arterioles in part through the suppression of sympathetic nerve activity in Dahl salt-sensitive rats [22]. Concordantly, Nakamura *et al* reported that azelnidipine significantly decreased albuminuria and the markers for oxidative stress, and alleviated the renal tubular injury in patients with diabetic nephropathy [23]. 

## CLINICAL STUDIES USING VARIOUS CCBs

A multi-center prospective study (including our institution) was conducted to assess the effects of efonidipine on renal function in hypertensive patients with CKD [24]. Patients were divided into two groups, i.e., an efonidipine-treated group and an ACE inhibitor-treated group. The results of the study showed almost similar hypotensive effects in the efonidipine- and the ACE inhibitor-treated group over the course of 2 years. Of interest, urinary protein excretion was decreased in a similar manner in both the groups. Furthermore, when the efonidipine-treated patients were categorized based on the mean blood pressure achieved at the end of the study (i.e., ≤100 mmHg and >100 mmHg), the urinary protein excretion was equally reduced in both subgroups. This finding lends support to the possibility that efonidipine exerts renoprotective action independent of systemic blood pressure. 

In JATOS trial, a large-scale clinical trial evaluating the optimal target blood pressure in elderly hypertensive patients, the renal sub-analysis showed that efonidipine not only prevented the decrease in eGFR in patients with eGFR < 60 ml/min but also improved eGFR [25] in patients with proteinuria. Furthermore, a prospective cohort study attempting a direct comparison of renal effects of T-type and L-type CCBs in non-diabetic CKD patients reveals more beneficial effects of T-type CCBs than L-type CCBs on renal endpoints [26]. 

CARTER trial evaluated the effects of cilnidipine and amlodipine on urinary protein excretion in patients with CKD already treated with renin-angiotensin system inhibitors [27]. This trial directly comparing the proteinuria-reducing effect showed cilnidipine reduced proteinuria whereas amlodipine had no effect or rather increased proteinuria. 

In contrast, L-type CCBs have been shown to decrease GFR in AASK [28]. In the renal subanalysis of CASE-J trial, the amlodipine-treated group manifested greater numbers of renal event (i.e., doubling of serum creatinine or end-stage renal failure) than the candesartan-treated group [29]. 

## FUTURE PERSPECTIVES

The current understanding of the effects of CCBs indicates that the renal action of CCBs differs, depending on the CCB subtypes. Based on renal hemodynamic aspects, T-type and N-type CCBs are considered beneficial for glomerular pressure and subsequent renoprotection. Furthermore, the sympathetic nerve inhibition by azelnidipine and cilnidipine would be anticipated to offer additional beneficial effects in the protection of renal injury. It requires additional investigations to establish whether the CCBs with inhibitory activity on various Ca channel subtypes offer beneficial effects on renal outcomes.
